# Social Withdrawal and Romantic Relationships: A Longitudinal Study in Early Adulthood

**DOI:** 10.1007/s10964-021-01469-1

**Published:** 2021-07-12

**Authors:** Stefania A. Barzeva, Jennifer S. Richards, Wim H. J. Meeus, Albertine J. Oldehinkel

**Affiliations:** 1 Department of Psychiatry, Interdisciplinary Center Psychopathology and Emotion Regulation, University of Groningen, University Medical Center Groningen, Groningen, The Netherlands; 2 Research Center Adolescent Development, Utrecht University, Utrecht, The Netherlands

**Keywords:** Dating, Early adulthood, Late adolescence, Longitudinal, Relationship quality, Romantic relationships, Social withdrawal

## Abstract

Involvement in romantic relationships is a salient developmental task in late adolescence and early adulthood, and deviations from normative romantic development are linked to adverse outcomes. This study investigated to what extent social withdrawal contributed to deviations from normative romantic development, and vice versa, and the interplay between withdrawal and couples’ relationship perceptions. The sample included 1710 young adults (55–61% female) from the Tracking Adolescents’ Individual Lives Survey cohort and their romantic partners. Data were collected across 4 waves, covering romantic relationships from ages 17 to 29 years. The results showed that higher withdrawal predicted a higher likelihood of romantic non-involvement by adulthood, consistently being single at subsequent waves, and entering one’s first relationship when older. Withdrawal moderately decreased when youth entered their first relationship. Male’s withdrawal in particular affected romantic relationship qualities and dynamics. These results provide new insights into the developmental sequelae of withdrawn young adults’ romantic relationship development.

## Introduction

Developmental Task Theory (Roisman et al., 2004) posits that involvement in romantic relationships becomes a salient developmental task during late adolescence and early adulthood, and delaying or not meeting this task is related to adverse outcomes. Indeed, romantic relationships become increasingly prevalent throughout these ages, with over half of adolescents having been romantically involved by the age of 16, and the large majority by early adulthood (Carver et al., 2003). Romantic relationships not only become more common, but also shift in their qualities and functions across these ages: they become longer-lasting and a main source of support and intimacy (Collibee & Furman, 2015; Lantagne & Furman, 2017). A comprehensive investigation of the specific features of romantic relationship development that withdrawal affects and is affected by has been lacking in the extant literature. This study focused on the longitudinal associations between social withdrawal and romantic relationships in late adolescence and early adulthood. Specifically, it investigated to what extent withdrawal contributed to deviations from normative romantic development and the interplay between withdrawal and couples’ perceptions of the quality of relationships. Three key features were examined as indicators of romantic development: involvement (lifetime involvement, current involvement, and timing), relationship quantity (number of partners and relationship duration), and relationship quality (commitment, satisfaction, support, and conflict; Collins, 2003).

Social withdrawal is an umbrella term referring to the voluntary self-isolation from familiar and unfamiliar others through the consistent display of solitary behaviors, such as avoiding social interaction and spending excessive time alone (Rubin et al., 2009). The motivation to withdrawal varies between individuals and differentiates three types of withdrawal: shyness, unsociability, and avoidance (Coplan & Armer, 2007; Ozdemir et al., 2015). Phenotypic withdrawal behaviors overlap across these withdrawal types. The current study uses the term “social withdrawal” to refer to the global, multidimensional, behavioral phenotype of voluntary self-isolation. Previous studies using this global conceptualization have indicated that 12 to 23% of individuals are persistently withdrawn throughout adolescence, and that withdrawn adolescents report less social affiliation, social contact, and social competence, and more anxiety than their non-withdrawn peers (Barzeva et al., 2019a; Tang et al. 2017). The romantic development of withdrawn adolescents and young adults may differ from the normative patterns due to deviations in previous social experiences that are usually conducive to romantic involvement, such as friendships (Kingery et al., 2010). The sequential stage theory of heterosexual romantic relationship emergence states that youth develop social-emotional competencies in the context of same-sex friendships in childhood, and start to apply these competencies in mixed-sex peer groups during adolescence (Connolly et al., 2004; Connolly et al., 2000). Mixed-sex groups provide opportunities to learn how to approach and interact with opposite-sex peers, who are also potential dating partners. Withdrawn youth, however, do not follow this cascade of development (Nelson et al., 2008). They experience difficulties initiating and maintaining same-sex friendships, subsequently leading to a smaller mixed-sex peer group. Not participating in a mixed-sex peer group hinders the development of important social skills for romantic relationship initiation and maintenance, and limits the size of adolescents’ dating pool.

Withdrawn youth’s formative experiences may set the stage for non-normative or delayed romantic relationship involvement and quantity in late adolescence and beyond (Raley et al., 2007; Shulman & Connolly, 2013). That is, having had fewer opportunities to learn how to approach and interact with opposite-sex peers in adolescence (Barry et al., 2013; Nelson et al., 2008) is likely to contribute to anxiety, rejection sensitivity (Gazelle & Druhen, 2009), and avoidance of novel social situations, such as when asking someone out on a date, and hence to delay romantic involvement. While this is a provoking notion, the empirical work on withdrawn individuals’ romantic involvement has been scarce, has mainly concerned the shyness aspect of withdrawal, and shows somewhat mixed findings. A seminal study of shy children born in the 1920s found that men, but not women, who were shy as children were older than their non-shy counterparts when they first married (Caspi et al., 1988). More recent studies have found that withdrawn and inhibited individuals become romantically involved at an older age than their more sociable peers (Boisvert & Poulin, 2016; Meeus et al., 2011), and those who were currently involved were less shy than those who were not (Roswell & Coplan, 2013; with an exception in Schmidt et al. 2017). The relations between withdrawal and romantic relationship quantity have never been tested directly, but one study found that shy young adults date less frequently (Leck, 2006), suggesting a possible effect of withdrawal on the quantitative features of romantic relationships, such as the number of partners and the duration of relationships. If withdrawn youth likewise date less frequently, it would suggest that they change partners less often, leading to having fewer partners with possibly longer relationship durations.

Entering a romantic relationship for the first time might have an effect on youth’s withdrawal. Developmental Task Theory suggests that when life events are off-time—occurring earlier or later than the majority of peers—adolescents experience negative social sanctions for deviating from the normative pattern of development and receive fewer social resources from peers (Furman & Collibee, 2014). When adolescents’ life events become normative in the context of their peers’ experiences, social sanctions may be lifted and more social resources provided, leading to greater socio-emotional adjustment. Additionally, the first romantic relationship is unique and of particular importance to young adults’ socio-emotional adjustment because it represents a shift in social identity, namely as a “girlfriend” or “boyfriend” (Raley et al., 2007). This new identity, and the social roles and experiences that come with it, changes how young adults perceive themselves and are perceived by others; when entering a romantic relationship for the first time, young adults feel an increase in autonomy, status, belonging, and social support, and may be seen by others as more mature and as a potential dating partner (Raley et al., 2007). These provisions may lead to a decrease in social withdrawal. Although an intriguing proposal, the effect of reaching the developmental task of romantic involvement on youth’s withdrawal has not been tested yet.

Despite the possible decrease of withdrawal when entering a romantic relationship for the first time, withdrawn adolescents and young adults may differ from their non-withdrawn peers in the qualities of their romantic relationships. By late adolescence and early adulthood, individuals are better equipped with relationship maintenance skills and start seeking and entering longer-lasting, more committed, supportive, and exclusive relationships than early adolescents (Shulman & Connolly, 2013). Starting from about 17 years of age, young adults transition from more sporadic romantic relationship involvements that resemble friendships to more intimate and committed relationships with a greater dyadic orientation (Seiffge-Krenke, 2003; Shulman & Connolly, 2013). In turn, being involved in a committed romantic relationship is linked to a decline in emotional problems in early adulthood (Meeus et al., 2007). The transition to more committed relationships may occur later for withdrawn young adults, who have less romantic experience due to delays in romantic involvement. Thus, higher withdrawal might predict lower commitment in young adults’ romantic relationships, and lower commitment, in turn, predict higher withdrawal. Furthermore, it is well documented that the formative qualities of adolescent friendships are linked to concurrent and future romantic relationship qualities (Collins & Van Dulmen, 2006; Collins et al., 2009). Withdrawn youth tend to report friendships characterized by low support and high conflict (Rubin et al., 2006), which likely translates into less satisfying romantic relationships. Empirical studies on the effects of withdrawal on romantic relationship qualities are rare, however, and have predominantly focused on the effects of shyness on relationship satisfaction. These studies have consistently found that shy adolescents and young adults report lower satisfaction than more sociable individuals (Luster et al., 2013; Nelson et al., 2008; Roswell & Coplan, 2013; Tackett et al., 2013; except in Schmidt et al., 2017). This effect is speculated to occur because of withdrawn individuals’ social inhibition and rejection sensitivity, which leads to decreased responsiveness, self-disclosure, and intimacy with their romantic partners (Luster et al., 2013). These characteristics might likewise be related to support and conflict.

Another possible reason for the low romantic relationship quality ratings of withdrawn youth are negative cognitive biases. When assessing their relationships, withdrawn individuals might report low quality because they are more attentive toward the negative aspects of their relationships and recall more negative interactions with their romantic partners (Gazelle & Duhen, 2009). In that case, their romantic partners might have more positive perceptions of the relationship. While untested in withdrawn young adults’ romantic relationships, these effects have been found in the romantic relationships of individuals with high attachment anxiety, which is characteristic of withdrawn individuals’ attachment style (Roswell & Coplan, 2013; Rubin et al., 2009). Anxiously attached individuals perceive more conflict and less support in their romantic relationships than securely attached ones, and these biased perceptions are associated with greater emotional distress and decreased satisfaction with and commitment to their romantic partners (Campbell et al., 2005). Thus, romantic partners’ ratings may provide additionally informative, less biased relationship quality information, and social withdrawal may have a differential effect on self- and partner-reported relationship perspectives, but empirical evidence has been lacking so far. In sum, how withdrawal directly affects self- and partner-reported romantic relationship qualities—commitment, satisfaction, support, and conflict—and if these relationship qualities affect withdrawal, has remained largely unknown.

Sex- and ethnicity-based social norms may contribute to differences between males and females and Western and non-Western youth in romantic relationship development and the effect of withdrawal. Although males and females may both experience non-normative romantic development, withdrawn males may be particularly affected because inhibited behavior violates gender-normative expectations of male dominance and assertiveness (Doey et al., 2014). Additionally, females may enter romantic relationships with more relationship maintenance skills than males. In childhood and adolescence, girls tend to prefer involvement in more intimate, dyadic relationships (Hall, 2011; Rose & Rudolph, 2006), which provide more opportunities than boys’ friendships to become comfortable self-disclosing, develop intimacy with others, and learn conflict resolution skills (Giordano et al., 2006). Hence, girls’ friendships resemble romantic relationships more closely than boys’ friendships. Because of this, boys – especially withdrawn ones who lack social experience in general – may be less prepared for maintaining intimate relationships than girls. Little is known about ethnic differences in social withdrawal and romantic relationship development, but some differences may be expected due to cultural norms around inhibited behavior (Chen & Tse, 2008; Coplan et al., 2012) and dating, especially in involvement and timing (Connolly & McDonald, 2020). Thus, sex and ethnicity were included as covariates in all models to adjust for possible confounding.

## The Current Study

There is reason to believe that social withdrawal is linked to deviations from normative romantic relationship development, and vice versa, but a direct empirical test of the extent to which withdrawn youth deviate from—and in which features of—normative romantic development has been lacking. The aim of this study was to investigate longitudinal associations between social withdrawal and romantic relationship development in late adolescence and early adulthood, a crucial life phase for romantic relationship-related exploration, decisions, and family formation. This was done, first, by testing to what extent withdrawal predicts deviations in romantic involvement (lifetime involvement, current involvement, timing) and relationship quantity (relationship length, the number of partners). Higher withdrawal was hypothesized to predict a greater likelihood of never having been involved in a romantic relationship by early adulthood, being single across late adolescence and early adulthood, initiating one’s first romantic relationship at an older age, and having longer-lasting relationships, but with fewer partners (Hypothesis 1). Second, this study tested if entering a romantic relationship for the first time changes one’s withdrawal levels. Withdrawal was expected to decrease after entering a romantic relationship for the first time, especially in individuals with high pre-involvement withdrawal (Hypothesis 2). A third set of tests addressed the question if withdrawal predicts deviations from romantic relationship qualities, and vice-versa, and examined the interplay between withdrawal and couple’s perceptions of their relationship. It was hypothesized that higher withdrawal will be concurrently and longitudinally associated with lower self-reported and partner-reported commitment, satisfaction, and support, and higher conflict, which in turn will be associated with higher withdrawal (Hypothesis 3).

## Method

### Participants

The study included 1710 participants from the prospective, population-based cohort Tracking Adolescents’ Individual Lives Survey (TRAILS; www.trails.nl) and their romantic partners, if applicable (*N* = 463 to 559 partners per assessment wave). The TRAILS sample was recruited from rural and urban areas of the North of the Netherlands, and includes individuals born between 1989 and 1991. Data collection began in 2001 when the participants were approximately 11 years old. Assessments occurred 7 times, every 2 to 3 years, from ages 11 to 29 years, and 64–96% of the initial sample participated in subsequent assessment waves. More details about the TRAILS recruitment and assessment procedure have been reported elsewhere (De Winter et al., 2005; Huisman et al., 2008; Oldehinkel et al., 2015). The current study used data from the last four assessment waves of TRAILS (T4-T7), when participants were approximately 19, 22, 26, and 29 years old (55–61% female across waves). About 90% of participants were from an ethnically Dutch background. Table 1 shows the participant and partner demographics, and descriptive statistics of the romantic relationship characteristics at each wave, and Table 2 the correlations between variables.

**Table 1:** Participant, partner, and romantic relationship characteristics

	T4	T5	T6	T7
Participant characteristics				
* N* participants	1679	1634	1523	1223
Age, years *M* (*SD*)	19.1 (0.59)	22.3 (0.65)	25.6 (0.60)	28.9 (0.59)
Female %	55.3	55.1	56.3	61.2
Dutch %^a^	89.6	89.7	90.6	91.0
Lifetime involvement %	81.8	91.9	96.1	97.0
Current involvement %	53.1	64.4	72.3	71.2
Social withdrawal *M* (*SD*)	0.31 (0.34)	0.32 (0.36)	0.38 (0.37)	0.34 (0.36)
Partner characteristics				
* N* partners	–	559	463	479
Age, years *M (SD)*	–	24.0 (3.62)	27.2 (3.46)	30.4 (3.83)
Age range	–	16.8–47.3	18.8–43.2	19.5–53.3
Female %	–	38.3	38.0	42.0
Relationship %				
Married	–	3.4	11.4	22.8
Registered partnership	–	1.3	13.8	18.0
Cohabitating	–	35.1	47.7	41.5
Not cohabitating	–	58.5	27.0	17.7
Relationship characteristics M (SD)				
Number of partners	2.28 (1.70)	2.33 (1.47)	2.21 (1.28)	–
Relationship duration, months^b^	19.0 (14.8)	31.9 (24.5)	49.0 (33.6)	70.3 (43.7)
Commitment (participant)	5.82 (1.42)	6.27 (1.16)	6.52 (0.89)	–
Commitment (partner)	–	6.36 (0.95)	6.57 (0.82)	–
Satisfaction (participant)	6.19 (1.08)	6.30 (0.93)	6.32 (0.84)	–
Satisfaction (partner)	–	6.28 (0.89)	6.27 (0.86)	–
Support (participant)	–	3.50 (0.41)	3.50 (0.48)	–
Support (partner)	–	3.36 (0.51)	3.43 (0.46)	–
Conflict (participant)	–	1.35 (0.34)	1.46 (0.39)	–
Conflict (partner)	–	1.51 (0.38)	1.50 (0.37)	–

^a^Other ethnicities include: Turkish, Moroccan, Surinam, Antillean, Indonesian or Mollucan, and Other not specified

^b^The average maximum relationship duration across participants was 54.9 months (SD = 38.8; range = 0.25–192). Scores could range from 1–10 for number of relationships, 1–7 for commitment and satisfaction, 1–4 for support, and 1–3 for conflict. Dashes indicate that the data was not collected at the assessment wave.

**Table 2:** Correlations between variables

Variable	1	2	3	4	5	6	7	8	9	10	11	12	13	14	15	16	17	18	19	20	21	22	23	24
1 T4 Withdrawal																								
2 T5 Withdrawal	0.56^**^																							
3 T6 Withdrawal	0.49^**^	0.62^**^																						
4 T7 Withdrawal	0.44^**^	0.54^**^	0.60^**^																					
5 Timing (age)	0.08^**^	0.05	0.07^*^	0.03																				
6 Number of partners	−0.03	0.04	0.03	0.09^**^	−0.07^**^																			
7 Max length relation.	−0.08^**^	−0.09^**^	−0.13^**^	−0.13^**^	−0.36^**^	−0.15^**^																		
8 T4 S Commitment	−0.04	0.02	−0.001	−0.04	−0.16^**^	−0.06	0.19^**^																	
9 T5 S Commitment	−0.06	−0.09^*^	−0.08^*^	−0.4	−0.11^**^	−0.05	0.25^**^	0.24^**^																
10 T6 S Commitment	−0.07^*^	−0.08^*^	−0.14^**^	−0.12^**^	−0.12^**^	−0.04	0.16^**^	0.22^**^	0.30^**^															
11 T5 P Commitment	−0.04	−0.08	−0.05	−0.02	−0.05	−0.02	0.22^**^	0.21^**^	0.26^**^	0.13^**^														
12 T6 P Commitment	−0.04	−0.05	−0.02	−0.07	−0.02	−0.04	0.08	0.06	0.09	0.12^*^	0.41^**^													
13 T4 S Satisfaction	−0.12^**^	−0.04	−0.10^*^	−0.10^*^	−0.09^*^	−0.09^*^	0.14^**^	0.60^**^	0.10^*^	0.12^*^	0.07	0.03												
14 T5 S Satisfaction	−0.10^**^	−0.16^**^	−0.13^**^	−0.10^*^	−0.05	−0.03	0.16^**^	0.14^**^	0.71^**^	0.24^**^	0.19^**^	0.01	0.18^**^											
15 T6 S Satisfaction	−0.09^**^	−0.15^**^	−0.20^**^	−0.20^**^	−0.07	−0.01	0.07^*^	0.14^**^	0.21^**^	0.68^**^	0.14^**^	0.15^**^	0.15^**^	0.30^**^										
16 T5 P Satisfaction	−0.08	−0.12^**^	−0.15^**^	−0.08	0.03	−0.01	0.12^**^	0.13^*^	0.17^**^	0.13^*^	0.64^**^	0.33^**^	0.06	0.25^**^	0.24^**^									
17 T6 P Satisfaction	0.003	−0.02	−0.09	−0.12^*^	0.05	0.03	−0.03	−0.02	0.03	0.18^**^	0.36^**^	0.62^**^	0.01	0.01	0.31^**^	0.45^**^								
18 T5 S Support	−0.12^**^	−0.14^**^	−0.14^**^	−0.14^**^	−0.01	−0.04	0.17^**^	0.11^*^	0.34^**^	0.10^*^	0.12^**^	0.10	0.18^**^	0.37^**^	0.14^**^	0.12^**^	0.08							
19 T6 S Support	−0.08^*^	−0.12^**^	−0.16^**^	−0.19^**^	−0.05	−0.02	0.05	0.10^*^	0.18^**^	0.49^**^	0.04	0.06	0.14^**^	0.23^**^	0.58^**^	0.12^*^	0.16^**^	0.30^**^						
20 T5 P Support	−0.08	−0.12^**^	−0.07	−0.06	0.04	−0.01	0.06	0.16^**^	0.17^**^	0.05	0.37^**^	0.18^**^	0.18^**^	0.25^**^	0.10^*^	0.46^**^	0.22^**^	0.20^**^	0.08					
21 T6 P Support	−0.12^*^	−0.11^*^	−0.11^*^	−0.14^**^	0.05	−0.03	−0.02	0.12	0.10	0.14^**^	0.24^**^	0.31^**^	0.02	0.04	0.21^**^	0.32^**^	0.38^**^	0.12^*^	0.16^**^	0.42^**^				
22 T5 S Conflict	0.09	−0.13^*^	0.15^*^	0.22^**^	0.09	0.06	−0.24^**^	−0.10	−0.25^**^	−0.30^**^	−0.18^**^	−0.21^**^	−0.07	−0.36^**^	−0.25^**^	−0.24^**^	−0.24^**^	−0.31^**^	−0.27^**^	−0.06	−0.02			
23 T6 S Conflict	0.02	0.04	0.19^**^	0.18^**^	−0.02	0.10^**^	−0.04	−0.08	−0.10^*^	−0.25^**^	−0.07	−0.10^*^	−0.12^*^	−0.13^**^	−0.36^**^	−0.10^*^	−0.28^**^	−0.11^**^	−0.30^**^	−0.09	−0.15^**^	0.49^**^		
24 T5 P Conflict	0.01	0.11	0.04	0.05	0.04	0.13^*^	−0.14^*^	−0.07	−0.02	−0.15^*^	−0.30^**^	−0.41^**^	−0.11	−0.15^*^	−0.20^**^	−0.44^**^	−0.31^**^	−0.07	−0.20^**^	−0.15^*^	−0.12	0.55^**^	0.21^**^	
25 T6 P Conflict	−0.004	−0.03	0.06	0.12^*^	−0.10*	0.04	0.03	−0.07	0.03	−0.07	−0.10	−0.24^**^	−0.14^*^	−0.01	−0.25^**^	−0.23^**^	−0.39^**^	−0.03	−0.18^**^	−0.10	−0.22^**^	0.34^**^	0.57^**^	0.42^**^

Relation. = romantic relationship; S = self-reported; P = partner-reported

^*^p < 0.05, ^**^p < 0.01, ^***^p < 0.001

### Data Collection Procedure

The Dutch Central Committee on Research Involving Human Subjects approved the TRAILS study. Participants provided written consent at T4 to T7. Data was collected by means of online questionnaires at these waves. At T5 to T7, participants nominated their current romantic partner. Research assistants contacted romantic partners for participation. When the partners consented to participate, they were sent online questionnaires via email.

### Measures

#### Social withdrawal

Social withdrawal was assessed at T4 to T7 with the mean of five items from the *Adult Self-Report* (ASR; Achenbach & Rescorla, 2003) withdrawn scale. In a sample of adults over the age of 18 years, the ASR withdrawn scale had high test-retest reliability and correlated moderately positively with measures of anxiety and social introversion (Achenbach & Rescorla, 2003). The five items included were: *I would rather be alone than with others*; *I am secretive or keep things to myself*; *I refuse to talk*; *I am too shy or timid*; and *I keep from getting involved with others*. These items were selected based on face validity and previous research (Booth-LaForce & Oxford, 2008; Tang et al., 2017), and items have been found to be longitudinally measurement invariant in adolescence and early adulthood (Barzeva et al., 2019a, 2019b). Items were rated on a 3-point scale, with 0 = Not at all, 1 = A little or sometimes, and 2 = Always or often true, in the past 6 months. Cronbach’s alpha ranged from 0.67 to 0.72 at each wave. For scales with fewer than ten items, an internal reliability cutoff of α > 0.60 is considered acceptable (Loewenthal, 2004).

#### Lifetime and current relationship involvement

Lifetime romantic relationship involvement was assessed at T4 to T7 with the item, *Have you ever had a boyfriend or girlfriend?* (0 = no, 1 = yes). Current romantic relationship involvement was assessed at T4 to T7 with the item, *Do you have a girlfriend or boyfriend at the moment?* (0 = no, 1 = yes).

#### Timing of first relationship

The approximate timing of participants’ first romantic relationship in late adolescence was assessed at T4 by subtracting the duration of their current relationship from their age at T4. If a participant did not have a partner at T4, an item from an Events History Calendar asking if they had started a romantic relationship with someone in the last two years was used instead. Participants could report the month and year in which they started up to two romantic relationships, and the date of the earliest reported relationship was used to calculate their age at that time. For those who entered a relationship for the first time at T5 to T7, their age at entry was determined by subtracting the length of that first relationship from their age at that wave. Early romantic relationships that ended before the age of 17 years were not included, whereas those that continued through the age of 17 were. The timing variable therefore reflects the age at which participants entered a more serious, attachment-based romantic relationship for the first time.

#### Number of partners

The number of romantic partners that participants had had was assessed at T6 with the item, *How often in your life have you had a steady boyfriend or girlfriend?* (rated from 0 to 10 or more times).

#### Relationship duration

The duration of the current romantic relationship was assessed with the item, *How long have you had a relationship with your current partner?* (in months) at T4 to T7. The maximum reported duration across the four waves was used in the analyzes to account for relationships that continued throughout multiple assessment waves.

#### Commitment

Romantic relationship commitment was assessed with mean of the three-item Commitment subscale of the Investment Model Scale (IMS; Rusbult et al., 1998). Participants completed the IMS at T4 to T6, and their partners at T5 and T6. The three items were: *I am focused on the long-term future of my relationship*; *I want my relationship to last a very long time*; and *I want my relationship to continue forever*. Items were rated on a 7-point scale, from 1 = completely disagree to 7 = completely agree (T4-T6 *α*_*self-report*_ = 0.84, 0.91, 0.90; T5-T6 *α*_*partner-report*_ = 0.87, 0.87).

#### Satisfaction

Romantic relationship satisfaction was measured by the mean of two items of the IMS Satisfaction subscale. Participants completed the questionnaire at T4 to T6, and their partners at T5 and T6. The two items were: *I am satisfied with my relationship* and *My relationship gives me what I need in terms of intimacy, friendship, etc*. Although the subscale also included the item *My relationship is much better than the relationships of others*, alpha was higher when excluding this item due to a lower overall mean rating of this item compared to the other two, and weak correlations between this and the other two items. Items were rated on a 7-point scale from 1 = completely disagree to 7 = completely agree (T4-T6 *α*_*self-report*_ = 0.73, 0.78, 0.72; T5-T6 *α*_*partner-report*_ = 0.81, 0.73).

#### Perceived support

At T5 and T6, participants and their partners responded to items assessing to what extent they felt supported by their romantic partner in specific domains. Items included *Decisions about work or education*; *Problems with your health*; *Spending your free time and your social contacts; Practical things*; and *Personal matters that concern you*. Items were rated on a 4-point scale, from 1 = no support, to 4 = a lot of support from partner (T5-T6 *α*_*self-report*_ = 0.72, 0.83, and *α*_*partner-report*_ = 0.82, 0.79). The scale represents the mean of the items ratings, with higher scores indicating more perceived support from one’s romantic partner.

#### Perceived conflict

Perceived relationship conflict was assessed at waves T5 and T6. Participants and their partners responded to five items about conflict situations occurring in the last 12 months, including: *Fierce discussions between you and your partner; One person blamed the other strongly; You didn’t talk to each other for a while; Fights got out of hand*; and *You no longer lived together (if applicable)*. Responses were given on a 3-point scale, where 1 = no, 2 = one time, and 3 = multiple times (T5-T6 *α*_*self-report*_ = 0.70, 0.72, and *α*_*partner-report*_ = 0.73, 0.70). The scale score represents the mean item ratings, with higher scores indicating more perceived relationship conflict.

### Statistical Analyzes

Analyzes were conducted in MPlus Version 80.4 (Muthén & Muthén, 1998–2017) using maximum likelihood with robust standard errors (MLR) estimation, unless otherwise stated. The analyzes were pre-registered on the Open Science Framework (www.osf.io/j6nrd), and an explanation of all deviations can be found in the Supplementary Materials. First, to test if social withdrawal predicted lifetime non-involvement, the age at one’s first relationship, maximum relationship duration, and the number of partners, an unconditional withdrawal latent growth curve model (LGCM), with correlated intercept and linear slope from T4 to T6 was specified. Sex and ethnicity were included as covariates, with paths to the withdrawal intercept and slope and to the outcomes. Paths from the withdrawal intercept and slope to the outcomes were specified. The path from the intercept to the outcomes shows how baseline withdrawal levels predicted the outcomes, and the path from the slope to the outcomes shows how the change in withdrawal between 19 and 29 years was associated with the outcomes. Additionally, a cross-lagged panel model (CLPM) with the concurrent and longitudinal effects between social withdrawal and current involvement at T4 to T7 tested if higher withdrawal consistently predicted being single across late adolescence and early adulthood. The CLPM was specified with stability paths of the same variable over time, the within-wave associations between withdrawal and current involvement, the cross-lagged paths from one variable at T_x_ to the other variable at T_x+1_, and a path from sex and ethnicity to T4 withdrawal and current involvement status. Good model fit was defined as a comparative fit index (CFI) > 0.90; standardized root mean residual (SRMR) < 0.06, and root mean square error of approximation (RMSEA) < 0.06.

Second, to test if entering a romantic relationship for the first time reduced adolescents’ withdrawal across 19 to 29 years, a dummy-coded a time-varying covariate (TVC), “entering first relationship,” with 0 = *has not yet entered a relationship* and 1 = *has entered first romantic relationship* (and TVC = 1 for all subsequent waves after entry) was used. Again, the withdrawal LGCM with sex and ethnicity as covariates was specified, now subsequently allowing the dummy-coded TVC to have an effect on withdrawal per time point. The WLSMV estimator was used due to the dichotomous nature of the TVC. A sensitivity analysis tested if the TVC effect was present within persons within a multilevel modeling framework. The difference between the original, SEM-based model and the multilevel model is that the multilevel model is a two-level (vs. single-level), univariate (vs. multivariate) model in which TVCs have random effect (vs. fixed effect) coefficients that vary over individuals (vs. over time). This means that in the sensitivity model, the effect of entering a romantic relationship for the first time on withdrawal is assumed to be constant across time, and the TVC effect on withdrawal represents the average shift in withdrawal when entering the first romantic relationship. Bayesian estimation with uninformative priors, 100 thousand iterations, and 2 Monte Carlo chains was used; the model stabilized at PSR = 10.001.

Third, to investigate the longitudinal, bi-directional associations between withdrawal and romantic relationship qualities, as reported by both the participants and their partners, a series of longitudinal actor-partner interdependence models (APIMs; Cook & Kenny, 2005) were performed, with data from 402 participants who had the same partner across T5 and T6. Six same-sex romantic dyads were excluded because of the sex grouping of the analyzes, explained below. Separate APIMS were tested for relationship commitment, satisfaction, perceived support, and perceived conflict; satisfaction is used to illustrate the modeling procedure. The APIM is designed to account for non-independence of observations within interpersonal relationships, with the romantic dyad as the unit of analysis instead of the individual. APIMs produce estimates of actor effects (how much a person’s satisfaction is predicted by their own prior satisfaction), and partner effects (how much a person’s satisfaction is predicted by their partner’s prior satisfaction). The traditional longitudinal APIM was extended in the current study by including participants’ T5 and T6 withdrawal to the model and using T5 withdrawal as both an actor and a moderating variable. To test if withdrawal moderated longitudinal actor and partner effects, all T5 predictors were centered, and two interaction terms were created, namely participant’s withdrawal by participant’s satisfaction, and participant’s withdrawal by partner’s satisfaction. To investigate differences between females and males, sex was specified as a grouping variable, thereby computing separate estimates for females and males within the same APIM framework. A grouping approach (instead of distinguishing actors and partners by sex) was necessary because withdrawal data for only one member per dyad was available. Actor effects were estimated by specifying paths from T5 to T6 participant’s satisfaction and T5 to T6 partner’s satisfaction. Partner effects were estimated by specifying paths from T5 participant to T6 partner satisfaction; and from T5 partner to T6 participant satisfaction. T6 participant and partner satisfaction were regressed on the two T5 interaction terms. T6 withdrawal was regressed on T5 withdrawal, participant and partner satisfaction, and the two interaction terms. All predictors were correlated, thereby estimating actor effects while controlling for partner effects, and vice versa. The residual variances of the outcome variables were also correlated to control for additional sources of non-independence. Paths from participants’ ethnicity and length of the romantic relationship (at T5) to all T5 predictors were added as controls. To correct for the false discovery rate (FDR) in multiple testing, the Benjamini and Hochberg (1995) method was applied. This method is more powerful and less conservative than the Bonferroni procedure. All observed *p*-values from the APIMs per sex (*n* = 84 for both females and males) were ranked, and alpha was specified as 0.05. The Benjamini-Hochberg adjusted *p*-value criterion was *p* < 0.009 for females and *p* < 0.014 for males. For simplicity, the criterion of *p* < 0.01 was used for all APIM effects.

## Results

### Social Withdrawal Predicts Romantic Relationship Involvement and Quantity

**Fig. 1:**
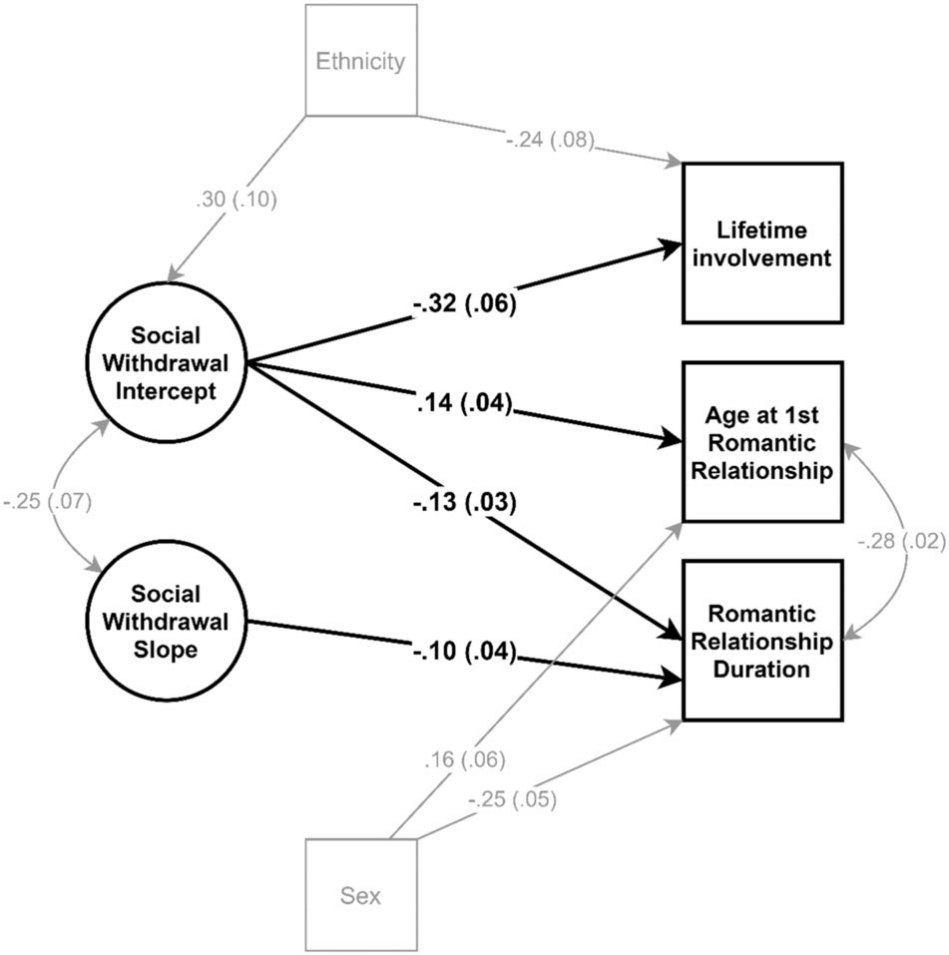
Significant paths from the Latent Growth Curve Model (LGCM), with the social withdrawal intercept and slope across 19 to 29 years predicting lifetime involvement, age at first romantic relationship, and maximum romantic relationship duration, controlling for ethnicity (0 = Dutch-origin, 1 = non-Dutch-origin) and sex (0 = female, 1 = male). No significant predictors of number of romantic partners emerged

Table 3 depicts the fit of all models. The LGCM (Fig. 1) indicated that having a higher withdrawal intercept significantly predicted a higher likelihood of never having been involved in a romantic relationship by early adulthood (*β* = −0.32, *p* < 0.001), becoming romantically involved at an older age (*β* = 0.14, *p* < 0.001), and having a shorter maximum romantic relationship duration (*β* = −0.13, *p* < 0.001). The withdrawal slope significantly predicted relationship duration (*β* = −0.10, *p* = 0.029); a steeper increase in withdrawal was associated with a shorter maximum relationship duration. Neither the withdrawal intercept nor the slope predicted the number of romantic partners. The model also showed that participants of non-Dutch origin were on average more withdrawn (*β* = 0.30, *p* = 0.005), and had a shorter maximum relationship duration (*β* = −0.24, *p* = 0.003) than Dutch-origin ones. Males were older at their first romantic relationship (*β* = 0.16, *p* = 0.010) and had a shorter maximum relationship duration (*β* = −0.25, *p* < 0.001) than females.

**Table 3:** Goodness-of-fit statistics of all structural equation models

Model	*n*	*χ*^2^	*df*	CFI	SRMR	RMSEA [95% CI]
Unconditional LGCM	1702	50.6	5	0.96	0.04	0.07 [0.06, 0.09]
TVC LGCM	1597	64.5	21	0.98	0.04	0.04 [0.03, 0.05]
Involvement CLPM	1710	217.3	24	0.91	0.05	0.07 [0.06, 0.08]
Commitment APIM	402	30.2	12	0.94	0.02	0.09 [0.05, 0.13]
Satisfaction APIM	402	7.8	12	1	0.01	0.00 [0.00, 0.05]
Conflict APIM	402	21.0	12	0.98	0.03	0.06 [0.00, 0.10]
Support APIM	402	14.3	12	0.99	0.02	0.03 [0.00, 0.08]

*n* = number of participants included in the analyzes; *CFI* = comparative fit index; *SRMR* = standardized root mean residual; *RMSEA* = root mean square error of approximation. *LGCMs* = latent growth curve models; *TVC* = time-varying covariate model (i.e. entering first romantic relationship); *CLPM* = cross-lagged panel model (current romantic relationship involvement); *APIM* = actor partner interdependence model. Good model fit for all models was determined by the following criteria: CFI > 0.90; SRMR < 0.06; RMSEA < 0.06

**Fig. 2:**
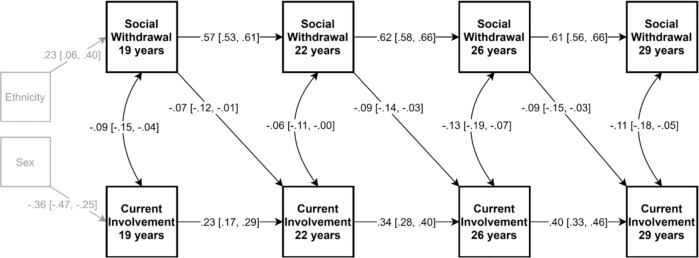
Significant effects in the Cross-Lagged Panel Model (CLPM) of social withdrawal and current romantic relationship involvement (0 = not currently in a relationship, 1 = currently in a relationship) across 19 to 29 years, controlling for ethnicity (0 = Dutch-origin, 1 = non-Dutch-origin) and sex (0 = female, 1 = male). 95% CI depicted in brackets

Figure 2 depicts the significant paths in the withdrawal and current involvement CLPM. The stability correlations of withdrawal at consecutive time points were strong and positive, and those of involvement were moderate and positive. Within-wave correlations between withdrawal and involvement were small and negative, indicating a decreased likelihood of current romantic involvement for more withdrawn youth. Cross-lagged paths were significant from withdrawal to current involvement status, but not vice versa. This means that across all ages, more withdrawn youth were less likely to have a romantic relationship approximately three years later, but relationship status, in turn, did not predict future withdrawal levels.

### The Effect of Entering a Relationship for the First Time on Withdrawal

The effects of the TVC “entering first romantic relationship” on withdrawal were significant and negative at all waves: T4 (*β* = −0.26, *p* < 0.001); T5 (*β* = −0.27, *p* < 0.001); T6 (*β* = −0.15, *p* = 0.043); T7 (*β* = −0.28, *p* = 0.008). This means that across all ages, participants who entered a romantic relationship for the first time were less withdrawn than those who had not yet become involved. These results replicated in the within-person effects of the multilevel model. The overall within-person effect of entering a romantic relationship for the first time on withdrawal was moderate and negative (*β* = −0.33, *95% CI* [−0.44, −0.21]), indicating a moderate decrease in withdrawal when an adolescent became involved for the first time.

### Longitudinal Within-Dyad Associations Between Withdrawal and Self- and Partner-Reported Relationship Qualities

Table 4 depicts all APIM effects. For females but not males, being in a romantic relationship for a longer time was associated with lower withdrawal and higher self- and partner-reported commitment. For males, ethnicity was associated with withdrawal, with non-Dutch-origin males reporting higher withdrawal than Dutch-origin males.

**Table 4:** Standardized estimates of the Actor-Partner Interdependence Models (APIMs) of participant social withdrawal and participant- and partner-reported commitment, satisfaction, support, and conflict, by participant’s sex

		Females				Males			
		Commitment	Satisfaction	Support	Conflict	Commitment	Satisfaction	Support	Conflict
Within-wave associations									
S_5_ ↔	P_5_	0.19	0.11	**0.24**	**0.43**	0.14	0.20^**+**^	0.10	**0.46**
SW_5_ ↔	S_5_	−0.06	−0.11	−0.08	0.06	−0.15	−0.24^**+**^	−0.05	−0.16
SW_5_ ↔	P_5_	−0.01	−0.07	−0.10	0.10	−0.05	−0.08	−0.21^**+**^	0.24
S_6_ ↔	P_6_	0.08	0.**32**	0.13	0.15	**0.30**	0.13	0.09	**0.51**
SW_6_ ↔	S_6_	−0.13^**+**^	−0.10	−0.07	**0.17**	−0.21	−**0.28**	−**0.25**	0.34
SW_6_ ↔	P_6_	0.02	−0.10	−0.01	0.10	−0.17	−0.12	0.12	0.16
Actor effects									
S_5_ →	S_6_	**0.34**	**0.24**	**0.37**	**0.52**	**0.40**	**0.40**	**0**.19^**+**^	**0.59**
P_5_ →	P_6_	**0.48**	**0.45**	**0.51**	**0.52**	**0.48**	**0.57**	**0.35**	**0**.18
SW_5_ →	SW_6_	**0.65**	**0.65**	**0.65**	**0.61**	**0.72**	**0.72**	**0.72**	**1.02**
Partner effects									
S_5_ →	P_6_	0.06	0.02	0.02	**0.25**	−0.06	0.01	0.15	0.32^+^
SW_5_ →	P_6_	−0.08	−0.02	−0.16	0.05	0.01	0.13	−0.13	0.07
P_5_ →	S_6_	−0.01	0.17^+^	−0.07	0.21^+^	0.08	0.21	−0.05	−0.20
SW_5_ →	S_6_	0.05	−0.01	0.02	0.04	−**0.26**	−**0.34**	−**0.33**	0.31
S_5_ →	SW_6_	0.01	−0.06	−0.07	**0.19**	0.04	0.07	−0.10	**0.33**
P_5_ →	SW_6_	0.04	0.01	0.02	−0.12	−0.10	**−0.29**	−0.03	−0.23
Interaction effects									
SW_5_*S_5_ →	S_6_	0.12	−0.01	−0.11	−0.01	**0.26**	0.11	0.05	0.19
SW_5_*P_5_ →	S_6_	−0.01	0.15	0.22	0.15	0.03	0.09	−**0.27**	−0.16
SW_5_*P_5_ →	P_6_	−0.004	0.04	−0.08	−0.02	−0.17	−0.18	−0.17	−0.24
SW_5_*S_5_ →	P_6_	0.03	−0.07	0.11	−0.12^+^	0.11	0.16^+^	−0.13	**0.43**
SW_5_*S_5_ →	SW_6_	−0.01	−0.06	−0.05	−0.04	−0.02	−0.02	−0.05	0.50
SW_5_*P_5_ →	SW_6_	0.04	0.09	0.02	0.13	0.03	−0.01	0.08	−**0.67**

S_5_ = self-reported relationship quality at T5; P_5_ = partner-reported relationship quality at T5; SW_5_ = self-reported social withdrawal at T5; S_6_ = self-reported relationship quality at T6; P_6_ = partner-reported relationship quality at T6; SW_6_ = self-reported social withdrawal at T6; Double-headed arrows indicate a correlational path and single-headed arrows a regression path

Bold coefficients are significant at *p* < 0.01; ^**+**^0.01 < *p* < 0.05

#### Actor and partner effects

For females and males, the longitudinal actor effects were moderate to strong for withdrawal and all self- and partner-reported relationship characteristics, with the exception of males’ partner-reported conflict and self-reported perceived support, which were not significant. This suggests that young adults’ withdrawal and relationship perceptions, and their partners’ relationship perceptions are moderately stable, controlling for ethnicity, relationship duration, and partner effects.

Seven significant out of 48 possible partner effects emerged across the APIMs (see Table 4); they showed a consistent pattern of the direction and magnitude of effects, providing confidence of robust (rather than chance) findings. Higher initial (T5) withdrawal predicted lower future (T6) self-reported commitment, satisfaction, and support for males, indicating that males who were initially more withdrawn reported decreased commitment to, satisfaction with, perceived support from their female partners. For both females and males, self-reported conflict predicted future withdrawal, and for females, self-reported conflict predicted partner-reported conflict. Partner-reported satisfaction also predicted future withdrawal for males.

#### Interaction effects

Simple slope estimates can be found in Table S1, and simple slope plots of the significant interaction effects in Figures S1-S5. Social withdrawal moderated the effect of T5 to T6 self-reported commitment in males: withdrawn males’ commitment to their female partners was more stable than non-withdrawn males’ commitment. Withdrawal also moderated several partner effects in males. First, it enhanced the association between males’ T5 partner- and T6 self-reported support: the less perceived support reported by the female partners of withdrawn males (> +0.50 *SD* average withdrawal), the less support these partners subsequently provided to the withdrawn males. For non-withdrawn males, the amount of support received by their partners did not predict future support from their partners. Second, withdrawal moderated the association from T5 self- to T6 partner-reported conflict in males. In withdrawn (> +0.50 *SD*) males, perceiving high levels of relationship conflict predicted more future partner-perceived conflict, whereas non-withdrawn (< 0.50 *SD*) males’ perceived conflict did not. Third, withdrawal moderated how partner-reported conflict predicted males’ future withdrawal: having a female partner that perceived high conflict predicted more withdrawal for non-withdrawn males (< −1 *SD*), and less withdrawal for highly withdrawn males (> +1.4 *SD*). There was no effect for males that were average on withdrawal.

## Discussion

Involvement in romantic relationships is a central developmental task of late adolescence and early adulthood (Roisman et al., 2004). Although previous work has suggested that withdrawn youth’s formative social experiences lead to delays in romantic development, empirical studies of the extent to which withdrawal contributes to deviations from normative romantic development, and in which specific features, were lacking. To address this gap, this study tested to what extent withdrawal predicts delays in romantic involvement and quantity; if entering a romantic relationship for the first time decreases withdrawal; and if withdrawal predicts self- and partner-rated romantic relationship qualities, and vice versa. Higher withdrawal across the decade of late adolescence and early adulthood predicted delays in all aspects of romantic involvement and a shorter longest-lasting romantic relationship, but did not influence the number of romantic partners. When an adolescent became romantically involved for the first time, withdrawal moderately decreased. Despite this decrease, withdrawal remained an important factor in couples’ relationship quality ratings, especially affecting males’ relationship perceptions and dynamics. These results provide insights into the developmental sequelae of withdrawn adolescents’ and young adults’ romantic relationship development.

As predicted, higher withdrawal was associated with a greater likelihood of never having been romantically involved, entering a romantic relationship for the first time when older, and a greater likelihood of being single three years later across all ages. These results corroborate findings from previous cross-sectional studies (Roswell & Coplan, 2013), and additionally indicate that withdrawn individuals’ romantic involvement delays are long-lasting, and that differences between withdrawn and non-withdrawn youth persist into adulthood. The delay of romantic involvement is likely attributable to withdrawn youth’s deviation from the formative cascade of development from same-sex friendships to mixed-sex peer groups to romantic partnerships (Nelson et al., 2008). Having had fewer opportunities to learn how to approach and interact with opposite-sex peers in adolescence, withdrawn youth might be particularly anxious in novel romantic situations, or avoid them altogether (Barry et al., 2013; Gazelle & Druhen, 2009). Contrary to expectations that withdrawn young adults would have longer-lasting relationships with fewer partners (a hypothesis that was based on limited research; Leck, 2006), higher and increasing withdrawal across the early adulthood decade predicted a shorter maximum romantic relationship duration, and had no effect on the number of romantic partners. The effect of withdrawal on relationship duration may be due to the fact that withdrawn adolescents were older when they first became romantically involved, leading to comparatively less possible time for their relationship to have lasted by the point of assessment than non-withdrawn young adults’ relationships. It is also probable that the romantic relationships of withdrawn young adults are more likely to break-up than those of non-withdrawn young adults, leading to shorter-lasting relationships. Because of the additional socio-emotional costs of romantic relationship dissolutions, an empirical test of this latter possibility is warranted.

It is important to note that, while withdrawn and non-withdrawn young adults have diverging patterns of romantic involvement, it is not suggested that withdrawn youth *should* be involved, especially if they do not want to be. Postponing romantic involvement to older ages than their more sociable counterparts might even be adaptive for withdrawn individuals. Having more time before focusing on “settling down” with a romantic partner could provide the opportunity to develop in other domains in which withdrawn young adults are also delayed, such as identity development (Barry et al., 2013), selecting educational and career paths (Hamer & Bruch, 1997), reaching higher levels of education and income (Nelson et al., 2020; Schmidt et al., 2017), maximizing person-environment fit (Shulman & Connolly, 2013), and developing interpersonal skills in other social relationships (e.g. friends, classmates, colleagues). Catching up to their non-withdrawn peers in these domains could then contribute to better maintenance of and positive functioning in withdrawn young adults’ romantic relationships when they do emerge, and “increase the chances for better provision for the next generation” (Shulman & Connolly, 2013, p. 34). Withdrawn young adults who do desire a romantic relationship but feel unable to initiate contact with potential dating partners may nevertheless feel lonely, have low self-esteem, and subsequently withdraw further; hence attaining this goal and entering a romantic relationship for the first time can have marked benefits.

Indeed, young adults’ withdrawal decreased when they entered a romantic relationship for the first time across participants. Based on Developmental Task Theory, withdrawn adolescents and young adults experience negative social sanctions and receive less social support from their peers when they are delayed in becoming romantically involved (Furman & Collibee, 2014). When withdrawn individuals enter their first romantic relationships, they are no longer off-time in romantic involvement compared to their peers, and gain the new social identity of “girlfriend” or “boyfriend”. This shift in identity could lead withdrawn young adults to obtain more social status, belonging, and social support (Raley et al., 2007). Withdrawn young adults may be especially sensitive to this shift in identity because they are less socially integrated than non-withdrawn young adults. Especially for them, entering a relationship for the first time might lead to greater social integration, the development of interpersonal skills, decreased loneliness, increased self-esteem, and expansion of social networks via the romantic partners, subsequently decreasing withdrawal.

Despite the decrease in withdrawal when entering a romantic relationship for the first time, withdrawal remained a predictor and outcome of several unfavorable romantic relationship qualities. Reiterating the main within-couple findings, high withdrawal (1) predicted lower self-reported commitment, satisfaction, and support in males; (2) was predicted by higher self-reported conflict in males and females, higher partner-reported conflict in females, and lower partner-reported satisfaction in males; and (3) in males, was associated with interaction patterns in which partners who perceived less support subsequently provided less support; perceived relationship conflicts predicted partner-perceived relationship conflicts; and partner-perceived conflicts predicted low males’ future withdrawal. Taken together, these results indicate that males’ withdrawal plays a bigger role in the romantic relationship quality dynamics than females’ withdrawal. These results are consistent with the theory that the suboptimal romantic relationship qualities of withdrawn individuals are due to difficulties with self-disclosing, being responsive, and forming intimate bonds with romantic partners (Luster et al., 2013), but suggest that these mechanisms apply primarily to withdrawn males. Withdrawn males in particular may struggle to communicate, self-disclose, and form intimacy with their partners, subsequently leading to difficulties committing to their partner, feeling less satisfied in the relationship and fostering less satisfaction in their partner, providing and receiving less support, and perceiving more relationship conflict. Females’ withdrawal, in contrast, had no effect on self- or partner-reported romantic relationship qualities. These gender disparities may be due to the greater social acceptance of withdrawal in females, and females being better-prepared for romantic relationships than males. The child and adolescent literature suggests that withdrawn behaviors are less socially accepted in boys than in girls because inhibited behaviors are viewed as violations of gender-normative expectations of male assertiveness and dominance (Doey et al., 2014). This seems to apply to romantic partnerships in early adulthood as well: males appear to be more accepting of their withdrawn female partners than are females of their withdrawn male partners. Additionally, females may enter romantic relationships with more relationship maintenance skills than males. In childhood and adolescence, girls tend to prefer involvement in more intimate, dyadic relationships (Hall, 2011; Rose & Rudolph, 2006), which more closely resemble romantic relationships than boys’ friendships. Girls’ friendships provide more opportunities than boys’ friendships to become comfortable self-disclosing, develop intimacy with others, and learn conflict resolution methods (Giordano et al., 2006), skills which are conducive to maintaining intimate relationships. Highly withdrawn males may come to rely on their female partners to take primary responsibility of the social and emotional aspects of their romantic relationship. Tentative support for this idea is that the results indicated that female partner-perceived conflict predicted *less* future withdrawal in highly withdrawn males. A withdrawn male may struggle to communicate his feelings and needs to his partner when he feels unhappy in his relationship (Giordano et al., 2006)—subsequently contributing to other relationship problems and more withdrawal—but when his female partner feels unhappy, she may be better able to self-disclose, take initiative to resolve conflicts, and rebuild intimacy in the relationship (Giordano et al., 2006; Raley et al., 2007); skills learned in her friendships might contribute to relationship improvements and less withdrawal in her male partner.

Several limitations should be considered when interpreting these results. First, a broad conceptualization of social withdrawal was used, which did not assess underlying motivations to withdrawal such as fear of negative evaluation, social disinterest, or peer rejection. The various motivations for withdrawing may be associated with different patterns of romantic development. For example, unsociable-withdrawn youth have been found to have fewer difficulties initiating and maintaining friendships than anxious-withdrawn youth (Ladd et al., 2011), and may likewise have fewer difficulties initiating and maintaining romantic relationships. Yet, withdrawal subtypes overlap and withdrawn youth of any subtype tend to have worse social relationships than non-withdrawn ones (Eggum-Wilkens et al., 2020; Nelson, 2013). Regardless, future studies could investigate differences in how young adults from different withdrawal subtypes initiate and maintain their romantic relationships.

Second, only romantic relationships that did not end before the age of 17 years were investigated, and thus the analyzes did not account for involvement in earlier relationships in adolescence. The timing variable therefore reflects the age at which participants entered a more serious, attachment-based relationship for the first time. This possibly neglects formative romantic experiences that set the stage for more “adult-like” relationship functioning. In the context of withdrawal, this might not have confounded results heavily because withdrawn young adults were more likely to have never been romantically involved and entered romantic relationship when older, meaning that these later romantic relationships were probably their first ones. Nevertheless, it would be interesting to explore the effects of withdrawal on romantic relationships from early adolescence, when romantic interests are just emerging and involvement is more sporadic, to adulthood.

Third, partner reports were available across only two waves, which may limit the generalizability of the dyadic results to young adults who maintained the same partner across three or more years. Although the duration of these relationships was accounted for, young adults tend to engage in multiple relationships of various durations across early adulthood, which could not be captured. Future studies could test if results replicate across longer- and shorter-lasting romantic relationships throughout early adulthood.

Fourth, it was not possible to investigate to what extent both romantic partners’ withdrawal levels affected their relationship perceptions and each other’s withdrawal, because no data about partners’ withdrawal was available. This is a limitation because both partners’ withdrawal influences the quality of the romantic relationship, and there may be differential effects between couples with one withdrawn partner and those with both. Partners’ withdrawal levels may interact to predict relationship outcomes, for better or for worse. On the one hand, having similarly high withdrawal levels may contribute to greater mutual understanding and acceptance of one another’s inhibited behaviors within couples. On the other hand, withdrawal in both partners may lead to a lower quality relationship because both partners may be non-communicative and hinder intimacy development. The lack of partners’ withdrawal data also meant that the sample needed to be split by sex in order to investigate sex differences in within-dyad associations. This would not have been necessary if data on partners’ withdrawal were available, and not splitting the sample would have increased power to detect smaller effects. Grouping by sex also required the exclusion of same-sex couples, thus limiting the generalizability of the findings to heterosexual young adults. Also limiting the generalizability was that the sample included young adults from a predominantly Dutch background. Because there is cultural variation in romantic relationship development, ethnic differences in the associations between withdrawal and romantic relationship initiation and maintenance are likely and require more attention in future work.

Finally, several gaps that could not be addressed remain to be investigated in future studies. First, a replication of the finding that males’ withdrawal particularly affected their romantic relationship characteristics is needed. Previous reports of sex differences in the associations between withdrawal and romantic relationship characteristics have been somewhat mixed. Although there is a theoretical basis to expect that males – especially withdrawn ones – face more challenges in their romantic relationships than females, directly testing effects of possible mediators (e.g. intimacy, self-disclosure) would have provided more robust evidence of the proposed mechanisms underlying this association. Second, future studies investigating romantic relationships could include individuals’ desire for having a romantic partner, especially when assessing young adults, who have postponed romantic relationship-related decisions to increasingly older ages (Arnett, 2000; OECD 2017). It is likely that young adults, withdrawn and non-withdrawn, who do not particularly desire a romantic relationship fare better than those who do desire one but are not involved. Relatedly, investigating if there are benefits for withdrawn youth to delay romantic involvement for identity, educational, and career development warrants more attention. Finally, to obtain a more comprehensive picture of young adults’ romantic relationship development, interpersonal and dyadic behaviors across multiple romantic partners could be investigated. The large majority of the participants in this study changed partners across the late adolescence and early adulthood decade, but within-dyad dynamics could only be investigated within only one relationship. It would be interesting to see how social withdrawal affects romantic partner selection, if withdrawal affects relationship functioning in the same way across different partners, and how withdrawn individuals cope with romantic relationship dissolutions.

## Conclusion

The current study provided insights into the links between young adults’ withdrawal and romantic relationship development and a general theoretical framework which can be applied in future investigations into the complex social and romantic worlds of withdrawn young adults. This study investigated the longitudinal effects of social withdrawal on deviations from normative romantic development in late adolescence and early adulthood, and the interplay between withdrawal and couples’ relationship perceptions. The results indicated that withdrawal in late adolescence and early adulthood contributed to delays in romantic relationship involvement, and was associated with certain romantic relationship quantity and quality features. Withdrawn young adults became romantically involved when they were older, and were more likely to have never been involved by adulthood, likely due to their heightened anxiety and avoidance of novel social situations. There might be advantages to this delay, as withdrawn young adults have more time to catch up to their more sociable peers in other domains. Withdrawn youth who nevertheless initiated a romantic relationship for the first time benefited from it: they became less withdrawn. Entering a romantic relationship for the first time may improve social integration, interpersonal skills, socio-emotional functioning, and social networks. Regardless of this initial decrease in withdrawal, withdrawal affected the qualities of males’ romantic relationships, possibly due to males’ withdrawal being less socially accepted, and withdrawn males having particular difficulty communicating, self-disclosing, and building intimacy with their partners. Highly withdrawn males may come to rely on their female partners to take primary responsibility of the social and emotional aspects of their romantic relationship, perhaps because females tend to be more socialized for maintaining romantic relationships. Many of these proposed mechanisms remain to be directly and empirically tested. Because early adulthood is characterized by decisions about long-term commitments, including those in romantic relationships such as partner selection, cohabitation and marriage, and family formation, continued investigations into the effects of withdrawal on the developmental tasks of this period of life are warranted.

## Supplementary information

Supplementary Information
