# Supplementary material for: Social Withdrawal and Romantic Relationships: A Longitudinal Study in Early Adulthood
Source: J Youth Adolesc. 2021 Jul 12;50(9):1766–81. doi: 10.1007/s10964-021-01469-1 (PMC8352801; doi:10.1007/s10964-021-01469-1)
Supplement: Supplementary file 1 — Supplementary Information [file 10964_2021_1469_MOESM1_ESM.docx]

**Social Withdrawal and Romantic Relationships: A Longitudinal Study in Early Adulthood**

Supplementary Materials

**Table of Contents**

Pre-registration deviations**1**

RI-CLPM Results**2-3**

Table S1: APIM simple slopes4

Figure S1-S5: Simple slope graphs of significant APIM interaction effects**5-9**

**Pre-registration Deviations**

Several deviations from our original pre-registered plan should be mentioned. First, in addition to sex, we included ethnicity as a covariate because of possible differences between Western- and non-Western adolescents on both withdrawal and romantic relationship outcomes. Second, we planned to conduct hierarchical multiple regressions to investigate differences between adolescent and partners’ relationship perspectives in participants’ first reported relationship, but decided that this question would be better suited in a separate study due to the extensive nature of the present one. Hierarchical multiple regressions were never conducted. Third, we planned on running random-intercept cross-lagged panel models (RI-CLPMs) of romantic relationship support and conflict, but we discovered after pre-registering that the T7 data collection wave (which was ongoing at the time) did not assess these variables, nor commitment and satisfaction. Thus, we opted for longitudinal APIMs with T5 and T6 data. Additionally, we wanted to include partner-reported commitment and satisfaction as TVCs in the RI-CLPMs, but these variables were only available at two waves, and adolescent participants may have had the same or differing partners across time, leading to biased TVC estimates. While we ran the RI-CLPMs with only self-reported commitment and satisfaction, the results of these models are not reported in the main text because we realized that there was a major confounder in these models: 91% of young adults changed partners between T4 and T6. This means that the self-reported commitment and satisfaction variables were with regards to different partners at each assessment wave. For full transparency, we have reported the RI-CLPM model specification method and results here.

**RI-CLPM Specification**

First, we regressed each observed score on its own latent factor, with each loading constrained at 1, resulting in six latent factors, namely social withdrawal and self-reported commitment at three time points each. We specified the stability paths, within-wave associations, and cross-lagged effects between the latent withdrawal and latent commitment variables. Two random intercept factors were added to capture the between-person differences in withdrawal and commitment. The observed scores and indicators of these factors and all factor loadings were constrained to 1. The variances of the observed scores were constrained to zero, forcing all variance in the observed variables to be captured by the within- and between-person latent factor structure. The correlations between the overarching latent factors reflect how stable between-person differences in withdrawal and commitment are associated. The stability paths indicate the extent to which within-person deviations in withdrawal can be predicted by the adolescent’s prior deviation from their own scores. The T1 correlations indicate whether adolescents’ deviations from their own expected scores in one variable are linked to deviations from their own scores in the other. The within-wave correlations indicate if within-person changes in withdrawal are linked to within-person changes in commitment. The cross-lagged effects indicate whether within-person changes in one variable are predicted by deviations from their own expected scores on another variable, assessed one wave prior.

**RI-CLPM Results**

ICCs were .54 for social withdrawal (T4-T7); .21 for self-reported commitment (T4-T6); and .24 for self-reported satisfaction (T4-T6), indicating sufficient within-person variance to investigate within-person changes over time. Table A depicts the models’ fit and Table B the standardized estimates for all paths in the two RI-CLPMs. The overall between-person association between withdrawal and commitment was negative and small (*β* = -.18, *95% CI* [-.36, -.01]), and between withdrawal and satisfaction negative and moderate (*β* = -.34, *95% CI* [-.57, -.12]). In both models, the stability paths for withdrawal were small and positive, indicating that within-person deviations from an adolescent’s withdrawal at one wave were predicted by within-person deviations from one wave prior. There were no significant within-person stability paths for commitment and satisfaction, likely due to 91% of adolescents changing romantic partners at least once between T4 and T6. Within-wave associations between withdrawal and commitment and satisfaction were significant at T6 (*β_commitment_* = -.13, *95% CI* [-.25, -.02]; *β_satisfaction_* = -.14, *95% CI* [-.24, -.04]), meaning that within-person increases in withdrawal were associated with within-person decreases in commitment to and satisfaction with one’s romantic partner, and vice versa. No within-person cross-lagged effects between withdrawal and commitment emerged, but one cross-lagged path between withdrawal and satisfaction did: T6 satisfaction was inversely related to T7 withdrawal (*β* = -.17, *95% CI* [-.30, -.06]), meaning that within-person decreases in satisfaction at T6 predicted future increases in withdrawal.

As shown, there were not many significant within-person effects in the withdrawal and commitment and satisfaction RI-CLPMs, likely due to the fact that 91% of young adults changed partners over time. This suggests that commitment to and satisfaction with one’s partner is more partner-dependent than trait-like. Indeed, there were no within-person stability paths for self-reported commitment and satisfaction in these models, while there were significant actor (stability) effects in the APIMs that included the same partner across time. The only significant effects that emerged were at T6 and T7, which is probably because young adults are more likely to maintain the same romantic relationship over time, leading to more same-dyad pairs in later waves. These results point to the importance of investigating bi-directional associations across the multiple romantic relationships that young adults engage in, and focusing on longitudinal associations within romantic dyads rather than across individuals.

Table A

*Goodness-of-fit statistics of the RI-CLPMs*

| Model | *n* | ꭓ^2^ | *df* | CFI | SRMR | RMSEA [95% CI] |
| --- | --- | --- | --- | --- | --- | --- |
| RI-CLPMs |  |  |  |  |  |  |
| Commitment | 1,702 | 8.0 | 5 | 1 | .01 | .02 [.00, .04] |
| Satisfaction | 1,702 | 7.6 | 5 | 1 | .01 | .02 [.00, .04] |

Table B

*Standardized estimates of the within-wave, stabilities, and cross-lagged associations from the social withdrawal and self-reported commitment and satisfaction Random Intercept CLPMs*

| **Within-Wave Associations** | SW4 ⬄ R4 | SW5 ⬄ R5 | SW6 ⬄ R6 |  |  |
| --- | --- | --- | --- | --- | --- |
| Commitment | .001 | -.05 | -.13^*^ |  |  |
| Satisfaction | -.04 | -.09 | -.14^**^ |  |  |
| **Stabilities** | SW4🡪SW5 | SW5🡪SW6 | SW6🡪SW7 | R4🡪R5 | R5🡪R6 |
| Commitment | .16^**^ | .30^***^ | .29^***^ | .10 | .02 |
| Satisfaction | .17^**^ | .30^***^ | .27^***^ | .02 | .12 |
| **Cross-lagged Paths** | SW4🡪R5 | R4🡪SW5 | SW5🡪R6 | R5🡪SW6 | R6🡪SW7 |
| Commitment | .02 | .09 | -.01 | -.01 | -.09 |
| Satisfaction | .03 | .08 | -.08 | -.01 | -.18^**^ |

*Note*. SW = social withdrawal; R = romantic relationship characteristic in model (i.e. current involvement, commitment, or satisfaction). Double-headed arrows indicate a correlational path and single-headed arrows a regression path. ^*^*p* < .05, ^**^*p* < .01, ^***^*p* < .001

Table S1

*Actor-partner interdependence model simple slopes (by sex and withdrawal standard deviation)*

|  |  | *+/- SD SW* | *b* | *SE* | *p* |
| --- | --- | --- | --- | --- | --- |
| T5 partner-reported support 🡪  T6 self-reported support | Females | -1 | -.10 | .04 | .017 |
|  |  | -0.5 | -.07 | .04 | .058 |
|  |  | +0.5 | .004 | .04 | .907 |
|  |  | +1 | .04 | .04 | .362 |
|  | Males | -1 | .08 | .07 | .237 |
|  |  | -0.5 | .03 | .06 | .616 |
|  |  | +0.5 | -.08 | .04 | .043 |
|  |  | +1 | -.13 | .04 | .000 |
| T5 self-reported commitment 🡪  T6 self-reported commitment | Females | -1 | .19 | .12 | .107 |
|  |  | -0.5 | .22 | .10 | .032 |
|  |  | +0.5 | .29 | .08 | .000 |
|  |  | +1 | .32 | .08 | .000 |
|  | Males | -1 | .16 | .11 | .136 |
|  |  | -0.5 | .25 | .09 | .005 |
|  |  | +0.5 | .44 | .07 | .000 |
|  |  | +1 | .54 | .08 | .000 |
| T5 partner-report commitment 🡪  T6 partner-reported commitment | Females | -1 | .45 | .14 | .002 |
|  |  | +1 | .44 | .08 | .000 |
|  | Males | -1 | .28 | .07 | .000 |
|  |  | +1 | .14 | .04 | .002 |
| T5 self-reported conflict 🡪  T6 social withdrawal | Females | -1 | .08 | .03 | .013 |
|  |  | +1 | .06 | .03 | .026 |
|  | Males | -1 | -.01 | .04 | .860 |
|  |  | +1 | .25 | .08 | .002 |
| T5 partner-reported conflict 🡪  T6 social withdrawal | Females | -1.5 | -.10 | .04 | .016 |
|  |  | -1 | -.08 | .03 | .020 |
|  |  | +1 | -.01 | .03 | .825 |
|  |  | +1.5 | .01 | .04 | .798 |
|  | Males | -1.5 | .19 | .09 | .034 |
|  |  | +1 | -.26 | .13 | .056 |
|  |  | -1 | .10 | .06 | .082 |
|  |  | +1.5 | -.34 | .17 | .046 |
| T5 self-reported conflict 🡪  T6 partner-reported conflict | Females | -1 | .14 | .04 | .001 |
|  |  | +1 | .04 | .03 | .112 |
|  | Males | -1 | -.04 | .07 | .563 |
|  |  | +1 | .24 | .09 | .011 |
| T5 self-reported Satisfaction 🡪  T6 partner-reported satisfaction | Females | -1 | .19 | .16 | .236 |
|  |  | -0.5 | .10 | .11 | .344 |
|  |  | +0.5 | -.06 | .10 | .541 |
|  |  | +1 | -.14 | .14 | .305 |
|  | Males | -1 | -.38 | .19 | .048 |
|  |  | -0.5 | -.18 | .13 | .152 |
|  |  | +0.5 | .20 | .06 | .001 |
|  |  | +1 | .40 | .11 | .000 |


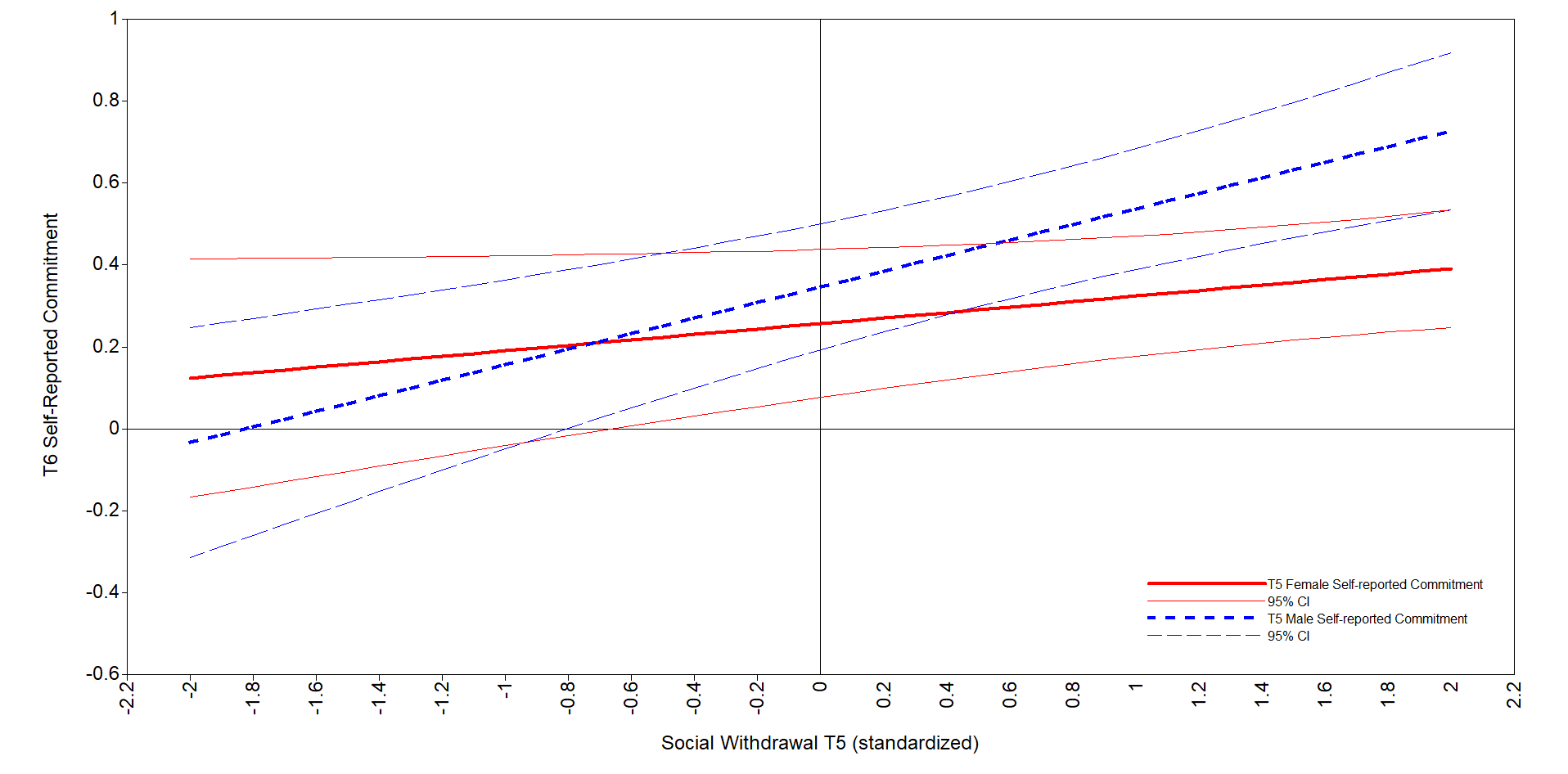


Figure S1. Simple slope graph of T5 withdrawal moderating the effect of T5 self-reported commitment on T6 self-reported commitment for females (solid red line) and males (dashed blue line).


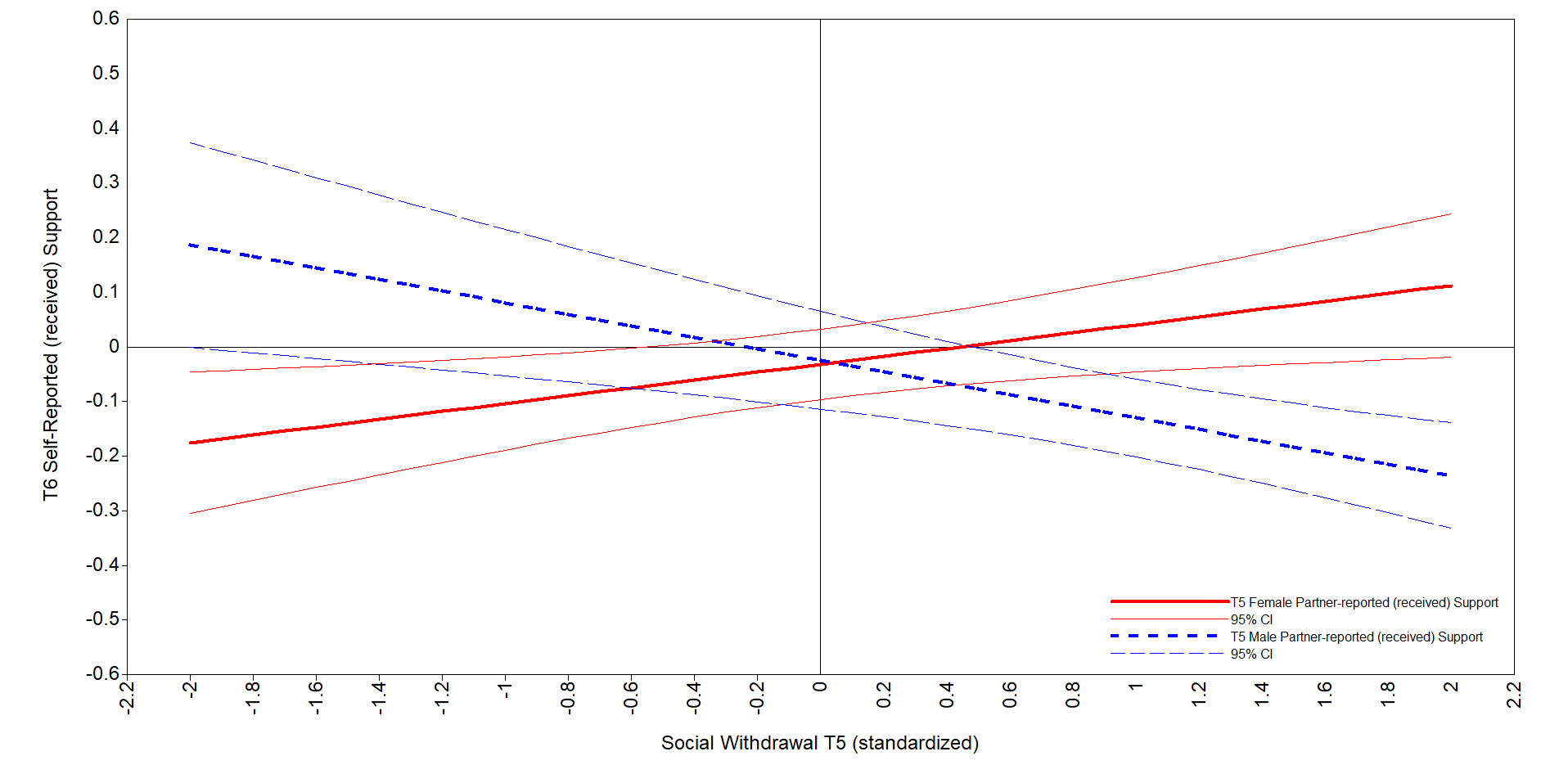


Figure S2. Simple slope graph of T5 withdrawal moderating the effect of T5 partner-reported (received) support on T6 self-reported (received) support for females (solid red line) and males (dashed blue line).


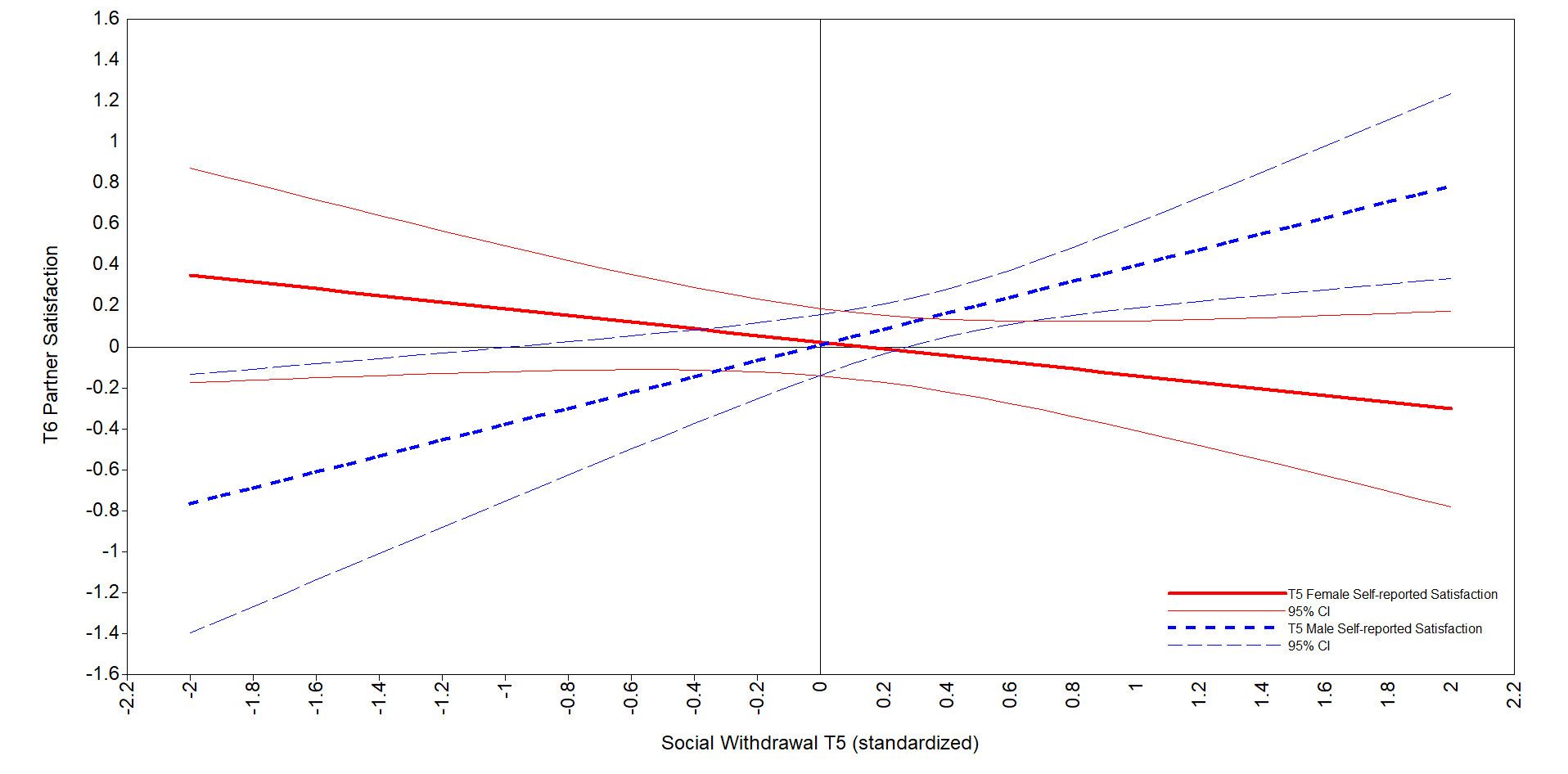


Figure S3. Simple slope graph of T5 withdrawal moderating the effect of T5 self-reported satisfaction on T6 partner-reported satisfaction for females (solid red line) and males (dashed blue line).


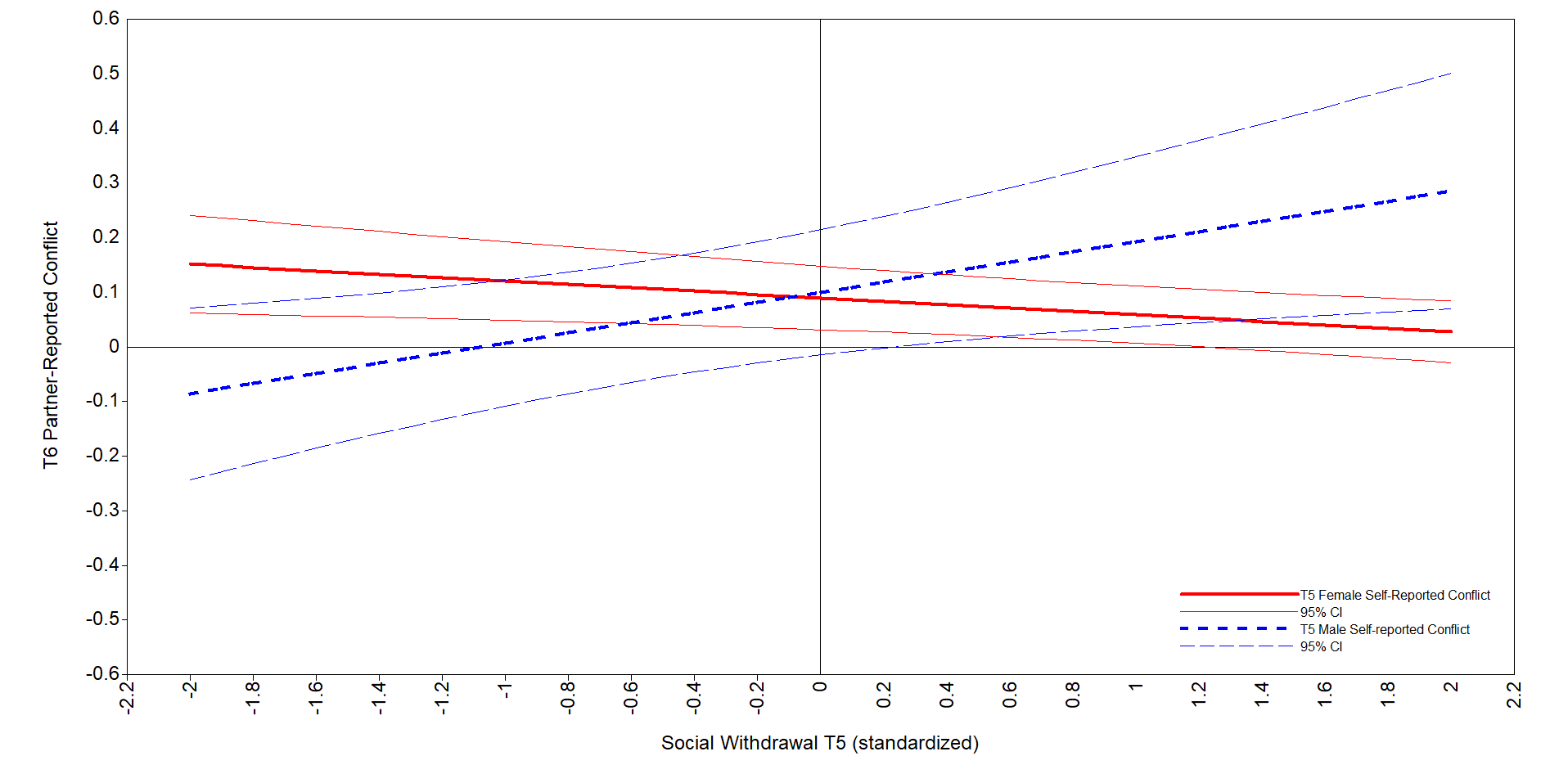


Figure S4. Simple slope graph of T5 withdrawal moderating the effect of T5 self-reported conflict on T6 partner-reported conflict for females (solid red line) and males (dashed blue line).


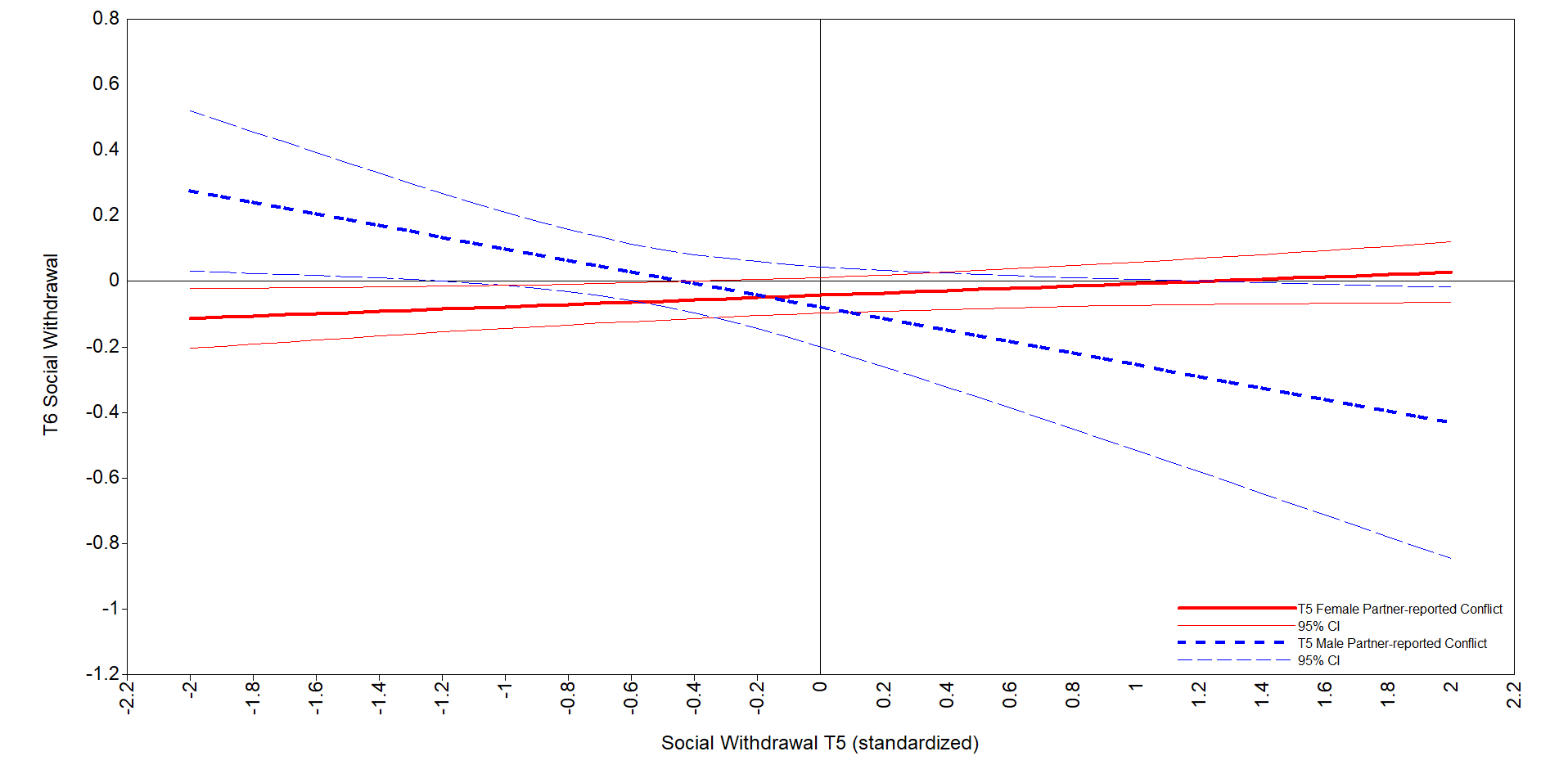


Figure S5. Simple slope graph of T5 withdrawal moderating the effect of T5 partner-reported conflict on T6 social withdrawal for females (solid red line) and males (dashed blue line).
